# Severity of Cognitive Impairment and Its Correlation With Depressive Symptoms and Influencing Factors in Elderly Patients With Chronic Diseases

**DOI:** 10.62641/aep.v54i2.2166

**Published:** 2026-04-15

**Authors:** Xing Liu, Wenxuan Ma, Xiaoyong Wei, Xiaoxia Yang, Xinli Ni

**Affiliations:** ^1^Department of Anesthesiology and Perioperative Medicine, General Hospital of Ningxia Medical University, 750004 Yinchuan, Ningxia, China

**Keywords:** elderly, chronic underlying disease, cognitive impairment, depressive symptoms, correlation

## Abstract

**Background:**

Elderly individuals frequently suffer from chronic underlying disease like hypertension and diabetes. These illnesses not only impair physical health but also show a close link to elevated rates of cognitive impairment and depressive symptoms. The mutual influence between these two issues can further diminish the quality of life of elderly patients. Nevertheless, there remains a shortage of systematic investigations into the cognitive-emotional relationship within this specific population.

**Methods:**

A total of 206 elderly patients with chronic underlying disease were enrolled retrospectively. Cognitive function was assessed using the Montreal Cognitive Assessment (MoCA). Depressive symptoms were evaluated via the 15-item Geriatric Depression Scale (GDS-15). To analyse the correlation between cognitive impairment and depressive symptoms, the Spearman correlation analysis, univariate analysis, and multivariate Logistic regression were employed.

**Results:**

Among the 206 patients, the primary chronic underlying diseases were hypertension (64.56%), diabetes mellitus (39.32%), coronary heart disease (27.67%), and chronic obstructive pulmonary disease (21.84%). Additionally, 45.15% of the patients had two or more chronic diseases. The prevalence of cognitive impairment stood at 49.51% (102 cases), while the prevalence of depressive symptoms was 40.78% (84 cases). No significant differences were observed in the prevalence of cognitive impairment and depressive symptoms among patients with different types of chronic diseases (all *p* > 0.05). Spearman correlation analysis revealed a significant negative correlation between MoCA scores and GDS-15 scores (r = –0.552, *p* < 0.001). Binary Logistic regression analysis indicated that factors such as body mass index ≥21.42 kg/m^2^, number of chronic diseases ≥2.5, GDS-15 score ≥4.5 points, and Multidimensional Scale of Perceived Social Support score ≥52.5 were independent risk factors for moderate-to-severe cognitive impairment (all *p* < 0.05).

**Conclusions:**

The severity of cognitive impairment in elderly patients with comorbid chronic underlying diseases increases with the exacerbation of depressive symptoms.

## Introduction

With the accelerated progression of global population aging, health issues among the elderly have attracted increasing attention. Elderly individuals are often affected by multiple chronic underlying diseases, such as diabetes mellitus, hypertension, cardiovascular diseases (CVD), and chronic obstructive pulmonary disease (COPD) [1]. These chronic diseases impose a substantial health burden on the elderly population. For instance, diabetes is one of the most common chronic non-communicable diseases among the elderly in China and Brazil [2]. Hypertension is a highly prevalent chronic disease worldwide [3]. CVDs are the leading cause of death in the elderly population [4]. COPD is the third leading cause of death in the United States and the fifth leading cause of disability worldwide [5].

These four chronic diseases are closely associated with the onset and progression of cognitive impairment and depressive symptoms. Long-term hypertension is recognised for its detrimental effects on cerebrovascular health, leading to reduced cerebral perfusion and subsequent cognitive decline [6]. CVDs and diabetes mellitus can induce cerebrovascular complications and neuroinflammation, thereby increasing the risk of cognitive impairment and depression [7, 8]. COPD exacerbates cognitive and emotional disturbances by causing chronic hypoxia that affects the structure and function of the central nervous system [9]. These chronic diseases not only severely impact the physical health and quality of life of the elderly but also bear a close connection to the occurrence of cognitive impairment and depressive symptoms [10]. Cognitive impairment encompasses various aspects, including memory decline, poor concentration, and reduced executive function. In severe cases, it can progress to dementia, imposing a heavy burden on patients and their families [11, 12]. Depressive symptoms are also common in the elderly population, manifested as symptoms such as low mood, loss of interest, and sleep disturbances, which further reduce the life satisfaction of elderly individuals [13].

Gaining an in-depth understanding of the relationship between the severity of cognitive impairment and depressive symptoms in elderly patients with chronic underlying disease holds great significance for formulating targeted intervention strategies and improving patient prognosis. At present, although some studies [14, 15] have focused on this field, research on the comprehensive impact of different chronic underlying disease and the specific mechanism of the association between cognitive impairment and depressive symptoms still needs to be improved. This study aimed to clarify the relationship between the severity of cognitive impairment and depressive symptoms in elderly patients with chronic underlying disease through a detailed analysis of 206 elderly patients over the past three years.

## Materials and Methods

### Study Subjects

This study has been approved by the Medical Research Ethics Review Committee of the General Hospital of Ningxia Medical University, with the ethical approval number: KYLL-2025-0271. All patients provided informed consent. The study was conducted in strict compliance with the Declaration of Helsinki [16].

This study employed a retrospective analysis method to extract clinical records and previous scale data from 206 elderly patients. All patients were consecutively enrolled at the General Hospital of Ningxia Medical University between July 2022 and June 2025, including both outpatients and inpatients.

Inclusion Criteria: Aged 60 years or older; diagnosed with at least one chronic disease, including hypertension, diabetes mellitus, CVD, and COPD; clear consciousness and ability to cooperate with the completion of relevant assessment scales.

Exclusion Criteria: Suffering from severe psychiatric disorders such as schizophrenia and mania; suffering from severe malignant tumours or other end-stage diseases.

### Study Methods

#### Collection of Chronic Condition Information

The medical records of patients were reviewed, and information such as the type, duration, and treatment status of their chronic underlying disease was recorded. Diseases were classified and diagnosed according to the International Classification of Diseases, 10th Revision (ICD-10) [17].

#### Cognitive Function Assessment

Data from the Montreal Cognitive Assessment (MoCA) [18] were extracted to evaluate patients’ cognitive function. This scale covers seven cognitive domains, including visuospatial and executive functions, naming, attention, language, abstraction, delayed recall, and orientation. It consists of 30 items, with 1 point assigned to each item, resulting in a total score of 30. For participants with less than 12 years of education, one additional point was added to their test results to adjust for educational level. The Cronbach’s α coefficient for the MoCA scale in this study was 0.75.

The cognitive function grading was assessed according to the following criteria. Cognitive function was classified according to the Chinese Guidelines for dementia and cognitive impairment in China: the diagnosis and treatment of mild cognitive impairment (2010 Edition) [19]. Considering the clinical characteristics of elderly patients with chronic underlying diseases as well as the sample size distribution of this study (the total proportion of patients with moderate-to-severe cognitive impairment was 29.41%, and the sample size of each group was less than 15% of the total sample when analysed separately), cognitive function was categorised into three levels according to severity. The specific criteria [20] are as follows: Normal cognitive function: Total MoCA score ≥26 (only used to define the baseline of “no cognitive impairment”, not serving as the core level for the “severity” analysis in this study); Mild cognitive impairment: 18 ≤ total score < 26; Moderate-to-severe cognitive impairment: Total score <18.

The criteria for assessing moderate to severe cognitive impairment in the merged population were based on the following theories. (1) Reference to authoritative guidelines: According to the Chinese Guidelines for the Diagnosis and Treatment of Cognitive Impairment and Dementia (2020 Edition) [21], patients with moderate and severe cognitive impairment exhibit high consistency in clinical outcomes (needing dependent care) and pathological mechanisms (comprehensive impairment of cognitive domains). (2) Basis from clinical practice: Among elderly patients with chronic underlying diseases, moderate and severe cognitive impairment are often associated with similar complication risks (e.g., falls, malnutrition, infections). The clinical intervention strategies for both groups mainly focus on “intensified care + symptomatic supportive treatment”, and there are no significant differences in their clinical management pathways.

The measures to ensure data authenticity are as follows. For patients with severe cognitive impairment (MoCA score <10), an assessment model combining “family caregiver proxy evaluation and researcher on-site observation” was adopted. Family caregivers were required to have provided daily care for the patients for more than 6 months. Prior to the assessment, they received training from researchers to clarify the scale’s criteria and scoring standards, and then completed the scale based on the patients’ daily performance over the past month. Meanwhile, researchers observed the patients’ responses to simple instructions (e.g., “point to your nose”, “pick up the cup”) and integrated cognitive function-related records from medical charts (e.g., previous Mini-Mental State Examination scores, head CT reports) for a comprehensive evaluation. The combination of family caregiver proxy evaluation results, researcher on-site observation findings, and medical chart records was used to determine the final cognitive function assessment result, ensuring it truly reflects the patients’ cognitive status [22].

#### Depressive Symptoms Assessment

The 15-item Geriatric Depression Scale (GDS-15) [23] was used for the assessment of patients’ depressive symptoms. This scale includes 15 questions, mainly covering aspects such as emotional state, hobbies, and sleep quality. The response uses a yes/no format. The total score is 15 points, and a score of ≥5 indicates the presence of depressive symptoms. The Cronbach’s α coefficient of this scale in the present study was 0.94.

#### Other Assessments and Tests

The short-form Activities of Daily Living (ADL) [24] scale was used to determine individuals’ ability to perform activities of daily living. The scale consists of six items, including dressing, bathing, eating, getting in/out of bed, using the toilet, and controlling urination and defecation. These items are categorised into four levels based on the degree of difficulty: “no difficulty”, “difficulty but able to complete independently”, “difficulty requiring assistance”, and “unable to complete”. Each item is assigned a score of 1 to 4 respectively, with the total score ranging from 6 to 24. The Cronbach’s α coefficient of this scale in the study was 0.82.

Social support was assessed by the Multidimensional Scale of Perceived Social Support (MSPSS) [25]. This scale comprises three dimensions: family support, friend support, and other support, with a total of 12 items. A 7-point Likert scale was adopted for scoring (1 = Strongly Disagree, 7 = Strongly Agree), and the total score ranges from 12 to 84. The Cronbach’s α coefficient of this scale was 0.93.

Nutritional risk was assessed using the Nutritional Risk Screening 2002 (NRS-2002) [26]. The total score is calculated by summing three components: disease severity score (0–3 points), nutritional status score (0–3 points), and age-adjusted score (0–1 point). The total score ranges from 0 to 7, with a total score of ≥3 indicating nutritional risk. The Cronbach’s α coefficient of this scale was 0.95.

High-sensitivity C-reactive protein (hs-CRP) levels were detected using the FAITH-1600 automatic biochemical analyser (Nanjing Laura Electronics Co., Ltd., Nanjing, China) based on the immunoturbidimetry method.

### Statistical Analysis

SPSS 22.0 (IBM Corporation, Armonk, NY, USA) statistical software was employed for data analysis. The normality of continuous data was verified using the Shapiro-Wilk test and Q-Q plots. Continuous data that conformed to a normal distribution were presented as mean ± standard deviation (x̄ ± s) and analysed using independent samples *t*-test; variables that did not conform to a normal distribution were expressed as P50 (P25, P75) and analysed via Mann–Whitney U test. Categorical data were displayed as numbers and percentages (%), and inter-group comparisons were performed using chi-square (χ^2^) test; Spearman correlation analysis was applied to explore the correlation between the severity of cognitive impairment and depressive symptoms.

Logistic regression analysis was used to identify the influencing factors of cognitive impairment severity. The optimal cut-off values for continuous variables were determined using the receiver operating characteristic (ROC) curve. The Youden index (sensitivity + specificity – 1) was calculated to identify the cut-off value that maximised the diagnostic accuracy of each variable. A two-side *p*-value < 0.05 was considered statistically significant.

## Results

### Baseline Data of Patients and Distribution of Chronic Underlying Disease

Among the 206 patients, there were 112 males (54.37%) and 94 females (45.63%). The age range was 60–83 years, with an average age of 68.65 ± 3.44 years. 133 patients (64.56%) had hypertension, 81 patients (39.32%) had diabetes mellitus, 57 patients (27.67%) had coronary heart disease, and 45 patients (21.84%) had COPD. Some patients suffered from multiple chronic diseases: 55 patients (26.70%) had two comorbid diseases, and 38 patients (18.45%) had three or more types. The average education levels of the patients were 8.18 ± 2.12 years, and the average disease duration was 6.92 ± 3.75 years. The total score of MoCA was 23.85 ± 5.84, and the total score of GDS-15 was 5.08 ± 1.82. (Table 1).

**Table 1.** **Baseline data of patients and distribution of chronic underlying disease**.

Characteristics		Data
Gender (male/female) [n (%)]		112 (54.37%)/94 (45.63%)
Age (years, $\bar{x}$ ± s)		68.65 ± 3.44
Body mass index (kg/m^2^, $\bar{x}$ ± s)		23.16 ± 2.52
Disease duration (years, $\bar{x}$ ± s)		6.92 ± 3.75
Education level (years, $\bar{x}$ ± s)		8.18 ± 2.12
Marital status [n (%)]		
	Married/cohabiting	125 (60.68%)
	Widowed/divorced/unmarried	81 (39.32%)
Living status [n (%)]		
	Living with family	139 (67.48%)
	Living alone/institutional care	67 (32.52%)
Smoking history [n (%)]		
	Yes	69 (33.50%)
	No	137 (66.50%)
Drinking history [n (%)]		
	Yes	70 (33.98%)
	No	136 (66.02%)
Number of chronic diseases (types, $\bar{x}$ ± s)		1.68 ± 0.88
Number of chronic diseases [n (%)]		
	1 type	113 (54.85%)
	2 types	55 (26.70%)
	≥3 types	38 (18.45%)
Chronic underlying disease [n (%)]		
	Hypertension	133 (64.56%)
	Diabetes mellitus	81 (39.32%)
	Coronary heart disease	57 (27.67%)
	COPD	45 (21.84%)
MoCA score ($\bar{x}$ ± s)		23.85 ± 5.84
GDS-15 score ($\bar{x}$ ± s)		5.08 ± 1.82

COPD, Chronic Obstructive Pulmonary Disease; MoCA, Montreal Cognitive Assessment; GDS-15, Geriatric Depression Scale-15.

### Comparison of Cognitive Impairment and Depressive Symptoms

**Fig. 1.**
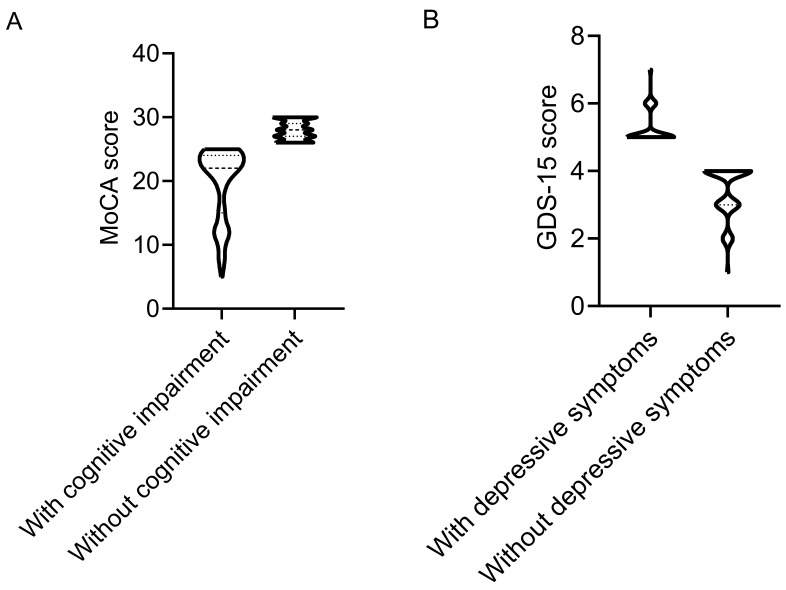
**Cognitive function and depressive symptoms of patients with and without cognitive impairment**. (A) Montreal Cognitive Assessment (MoCA) score. (B) Geriatric Depression Scale-15 (GDS-15) score.

Among all patients, 102 cases (49.51%) exhibited cognitive impairment, with a median (P25, P75) MoCA score of 22 (15, 24) points, whereas 104 cases (50.49%) had normal cognitive function, with a median (P25, P75) MoCA score of 28 (27, 29) points (Fig. 1A). Patients with cognitive impairment had a longer disease duration and higher GDS-15 scores, but lower MoCA scores, compared with those with normal cognitive function (all *p* < 0.05) (Table 2).

**Table 2.** **Comparison of baseline data between patients with and without cognitive impairment**.

Baseline data		Normal cognitive function (n = 104)	Cognitive impairment (n = 102)	t/χ^2^	*p*
Age (years, $\bar{x}$ ± s)		68.54 ± 3.53	68.75 ± 3.35	0.438	0.662
Gender [n (%)]				0.935	0.334
	Male	60 (57.69%)	52 (50.98%)		
	Female	44 (42.31%)	50 (49.02%)		
Number of chronic diseases (types, $\bar{x}$ ± s)		1.62 ± 0.85	1.75 ± 0.91	1.060	0.290
Chronic diseases [n (%)]					
	Hypertension	68 (65.38%)	65 (63.73%)	0.062	0.803
	Diabetes mellitus	36 (34.62%)	45 (44.12%)	1.949	0.163
	Coronary heart disease	26 (25.00%)	31 (30.39%)	0.748	0.387
	COPD	22 (21.15%)	23 (22.55%)	0.059	0.809
Education level (years, $\bar{x}$ ± s)		8.09 ± 2.06	8.28 ± 2.18	0.643	0.521
Disease duration (years, $\bar{x}$ ± s)		6.34 ± 3.43	7.48 ± 3.95	2.210	0.028
Number of chronic diseases [n (%)]					
	1 type	61 (58.65%)	52 (50.98%)	1.282	0.527
	2 types	26 (25.00%)	29 (28.43%)		
	≥3 types	17 (16.35%)	21 (20.59%)		
MoCA score (points, $\bar{x}$ ± s)		28.00 ± 1.46	19.63 ± 5.6	14.740	<0.001
GDS-15 score (points, $\bar{x}$ ± s)		3.68 ± 1.09	4.65 ± 0.91	6.713	<0.001

COPD, Chronic Obstructive Pulmonary Disease; MoCA, Montreal Cognitive Assessment; GDS-15, Geriatric Depression Scale-15.

Among all patients, 84 cases (40.78%) had depressive symptoms, with a GDS-15 score of 5 (5, 5.75), whereas 122 cases (59.22%) had normal cognitive function, with a GDS-15 score of 4 (3, 4) (Fig. 1B).

### Cognitive Impairment and Depressive Symptoms Across Different Chronic Underlying Disease

Among patients with hypertension, the prevalence of cognitive impairment and depressive symptoms was 48.87% (65/133) and 42.11% (56/133), respectively; among patients with diabetes mellitus, the prevalence of cognitive impairment and depressive symptoms was 55.56% (45/81) and 43.21% (35/81), respectively; for patients with coronary heart disease, the prevalence of cognitive impairment and depressive symptoms was 54.39% (31/57) and 50.88% (29/57), respectively; and in patients with COPD, the corresponding rates were 51.11% (23/45) and 44.44% (20/45), respectively. No statistically significant differences were observed in the prevalence of cognitive impairment (χ^2^ = 1.074, *p* = 0.783) or depressive symptoms (χ^2^ = 1.298, *p* = 0.730) across different chronic underlying disease (Table 3).

**Table 3.** **Cognitive impairment and depressive symptoms across different chronic underlying disease**.

Chronic underlying disease	Case	Cognitive impairment	Depressive symptoms
Hypertension	133	65 (48.87%)	56 (42.11%)
Diabetes mellitus	81	45 (55.56%)^a^	35 (43.21%)^a^
Coronary heart disease	57	31 (54.39%)^a⁢b^	29 (50.88%)^a⁢b^
COPD	45	23 (51.11%)^a⁢b⁢c^	20 (44.44%)^a⁢b⁢c^
χ ^2^		1.074	1.298
*p*		0.783	0.730

COPD, Chronic Obstructive Pulmonary Disease. ^a^indicates comparison with hypertension patients, ^b^indicates comparison with diabetes mellitus patients, ^c^indicates comparison with coronary heart disease patients, *p* > 0.05.

### Correlation Analysis Between Cognitive Function and Depressive Symptoms

**Fig. 2.**
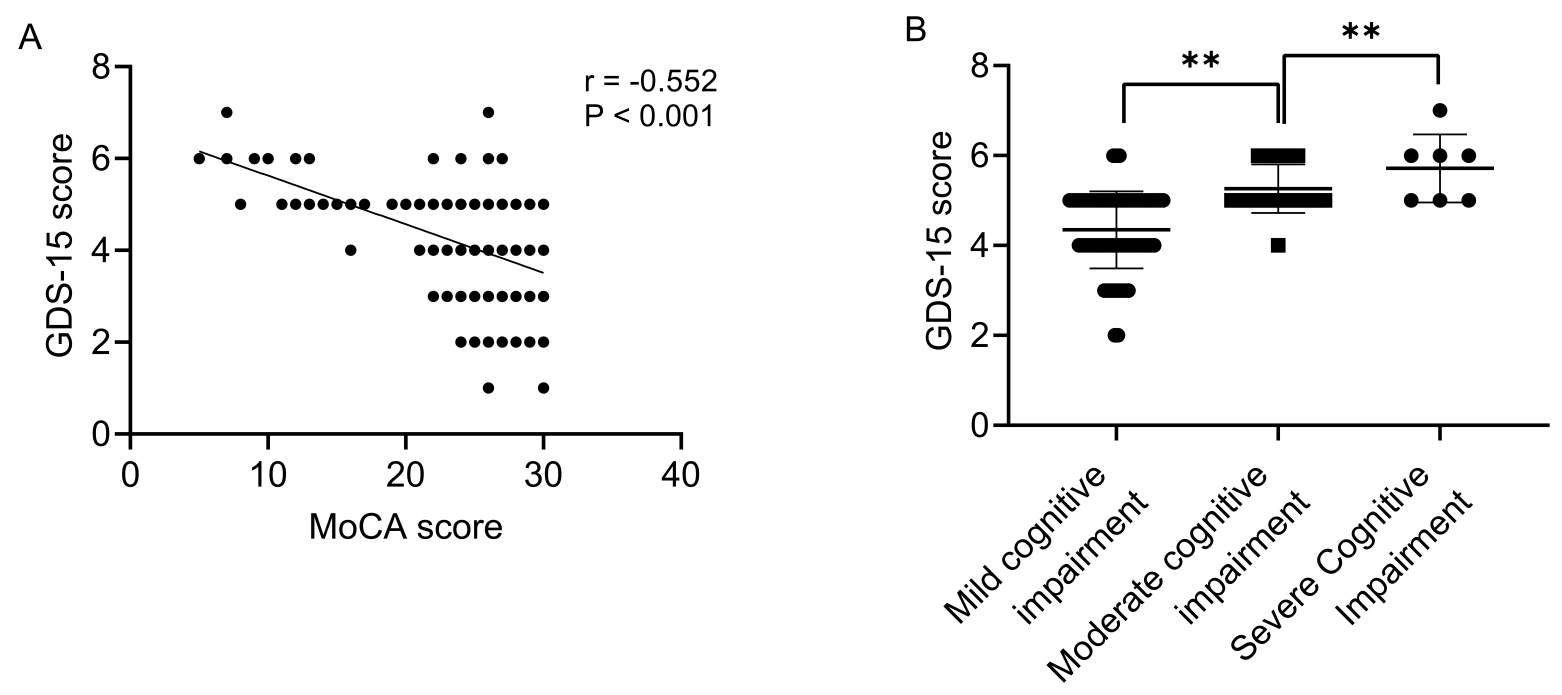
**Correlation analysis between cognitive impairment severity and depressive symptoms**. (A) Scatter plot of correlation between Montreal Cognitive Assessment (MoCA) score and Geriatric Depression Scale-15 (GDS-15) score; (B) GDS-15 scores across cognitive impairment severity. **p < 0.001.

To further explore the correlation between cognitive function and depressive symptoms beyond the initial binary classification, MoCA scores were treated as continuous variables for quantifying correlation strength, and cognitive impairment was further categorised into ordered subgroups (mild, moderate, severe) based on MoCA cut-offs to capture gradient relationships. Results of Spearman correlation analysis showed that MoCA scores had a significantly negative correlation with GDS-15 scores (r = –0.552, *p* < 0.001), indicating that the more severe the cognitive impairment, the more obvious the patient’s depressive symptoms. Patients were further divided into three groups based on the severity of cognitive impairment: mild (MoCA 18–25 points, 72 cases), moderate (MoCA 10–17 points, 23 cases), and severe (MoCA < 10 points, 7 cases). There was a significant difference in GDS-15 scores among different degrees of cognitive impairment (χ^2^ = 29.419, *p* < 0.001). Patients with moderate-to-severe cognitive impairment had higher GDS-15 scores with median of 5 (5, 6) points, while patients with mild cognitive impairment had lower GDS-15 scores with median of 5 (4, 5) points (Fig. 2).

### Univariate Analysis of Cognitive Impairment Severity

Among 102 elderly patients with cognitive impairment, 72 cases (70.59%) were in the mild group and 30 cases (29.41%) were in the moderate-to-severe group. Compared with the mild group, the moderate-to-severe group had a higher proportion of patients without a spouse, lower BMI and MSPSS scores, and a greater number of chronic diseases and higher GDS-15 score (all *p* < 0.05) (Table 4).

**Table 4.** **Univariate analysis of cognitive impairment severity**.

Influencing factors		Moderate-to-severe group (n = 30)	Mild group (n = 72)	t/Z/χ^2^	*p*
Age (years, $\bar{x}$ ± s)		68.23 ± 3.34	68.97 ± 3.33	1.022	0.309
Gender [n (%)]				3.484	0.062
	Male	11 (36.67%)	41 (56.94%)		
	Female	19 (63.33%)	31 (43.06%)		
Education level (years, $\bar{x}$ ± s)		8.40 ± 2.18	8.24 ± 2.18	0.338	0.736
Marital status [n (%)]				6.217	0.013
	With spouse (married/cohabiting)	12 (40.00%)	48 (66.67%)		
	Without spouse (widowed/divorced/unmarried)	18 (60.00%)	24 (33.33%)		
Living status [n (%)]				0.502	0.479
	Living with family	20 (66.67%)	53 (73.61%)		
	Living alone/institutional care	10 (33.33%)	19 (26.39%)		
Smoking history [n (%)]				0.174	0.677
	Yes	10 (33.33%)	21 (29.17%)		
	No	20 (66.67%)	51 (70.83%)		
Drinking history [n (%)]				0.989	0.320
	Yes	9 (30.00%)	15 (20.83%)		
	No	21 (70.00%)	57 (79.17%)		
BMI (kg/m^2^, $\bar{x}$ ± s)		22.09 ± 2.96	23.44 ± 2.28	2.489	0.014
Number of chronic diseases (types, $\bar{x}$ ± s)		2.30 ± 1.13	1.54 ± 0.69	4.155	<0.001
ADL score (points, $\bar{x}$ ± s)		17.07 ± 4.19	18.13 ± 3.90	1.224	0.224
NRS-2002 score (points, $\bar{x}$ ± s)		2.33 ± 0.94	2.11 ± 0.74	1.261	0.210
GDS-15 score		5.37 ± 0.60	4.35 ± 0.85	5.974	<0.001
MSPSS score (points, $\bar{x}$ ± s)		44.13 ± 12.62	62.99 ± 8.54	8.769	<0.001
hs-CRP (mg/L, $\bar{x}$ ± s)		3.45 ± 0.65	3.25 ± 0.91	1.092	0.278

BMI, Body Mass Index; ADL, Activities of Daily Living; NRS-2002, Nutritional Risk Screening 2002; GDS-15, Geriatric Depression Scale-15; MSPSS, Multidimensional Scale of Perceived Social Support; hs-CRP, high-sensitivity C-Reactive Protein.

### Logistic Regression Analysis of Cognitive Impairment Severity

Taking the severity of cognitive impairment as the dependent variable (mild = 0, moderate-to-severe = 1), and combining clinical significance and univariate analysis results (*p* < 0.05), indicators such as marital status, BMI, number of chronic diseases, depressive symptoms, and MSPSS score were included as independent variables in the binary Logistic regression model. The results showed that BMI ≥21.42 kg/m^2^, number of chronic diseases ≥2.5 types, GDS-15 score ≥4.5 points, and MSPSS score ≥52.5 points were independent risk factors for moderate-to-severe cognitive impairment in elderly patients with chronic underlying disease (all *p* < 0.05) (Table 5).

**Table 5.** **Logistic regression analysis of cognitive impairment severity**.

Variable	Assignment	B	SE	Walds	*p*	OR (95% CI)	Optimal cut-off value
Marital status	With spouse = 0, Without spouse = 1	0.635	0.819	0.602	0.438	1.887 (0.379, 9.390)	—
BMI	Actual value	–0.400	0.181	4.898	0.027	0.671 (0.471, 0.955)	21.42
Number of chronic diseases	Actual value	0.914	0.454	4.045	0.044	2.493 (1.024, 6.072)	2.5
GDS-15 score	Actual value	1.667	0.686	5.900	0.015	5.296 (1.380, 20.328)	4.5
MSPSS score	Actual value	–0.180	0.051	12.393	0.000	0.835 (0.756, 0.923)	52.5
Constant	—	8.030	6.438	1.556	0.212	3073.167	—

BMI, Body Mass Index; MSPSS, Multidimensional Scale of Perceived Social Support; OR, odds ratio; CI, confidence interval; GDS-15, Geriatric Depression Scale-15.

## Discussion

### Core Association Between Cognitive Impairment and Depressive Symptoms and Its Clinical Significance

This study demonstrates that, in the elderly population with chronic underlying disease, cognitive function (MoCA score) is significantly negatively correlated with depressive symptoms (GDS-15 score). This correlation remained stable even after controlling for confounding factors such as marital status, living arrangement, drinking history, BMI, and MSPSS score. These findings are highly consistent with the conclusions of multiple recent studies. For instance, Dai *et al*. [27], based on data from the Shanghai Brain Aging Study, reported a close correlation between cognitive impairment and depressive symptoms. Consistent with our data, a shared pathophysiological pathway—chronic disease-induced low-grade neuroinflammation disrupting cognitive and emotional regulatory networks—may underlie this comorbidity, providing a mechanistic basis for targeted interventions. From the perspective of clinical characteristics, the prevalence of cognitive impairment and depressive symptoms in this study was 49.51% and 40.78%, respectively, both higher than the levels in the general elderly population. According to a meta-analysis by Huang *et al*. [28], the prevalence of cognitive impairment and depressive symptoms in the community-dwelling elderly population is approximately 32.1% and 28.3%, respectively. This high prevalence may be related to the cumulative damage of chronic underlying disease to the neural-emotional regulatory system [29]. Multiple studies have confirmed that the co-occurrence of cognitive and emotional disorders in patients with comorbid diseases is significantly higher than that in patients without underlying diseases, making this population a high-risk group requiring key clinical attention [30].

### Independent Risk Factors for Cognitive Impairment and Mechanism Exploration

#### Cumulative Effect of the Number of Chronic Diseases

Logistic regression analysis showed that having ≥2.5 types of chronic diseases was an independent risk factor for moderate-to-severe cognitive impairment, which supports the theory of “cumulative comorbidity burden leading to superimposed cognitive risk”. Similar to the results of our study, a study by Liang *et al*. [31] confirmed that comorbidity (≥2 types of chronic diseases) is an independent risk factor for cognitive frailty, and patients with cognitive frailty have a significantly increased risk of adverse outcomes such as dementia and death, revealing a dose-response relationship between the number of chronic diseases and cognitive impairment. Consistent with this, 45% of our patients had ≥2 chronic diseases, with higher cognitive impairment prevalence, reinforcing the cumulative burden effect on cognition. The mechanism may be associated with neurotoxic cascading reactions caused by multi-system damage: cerebral small vessel disease induced by hypertension, accumulation of advanced glycation end products triggered by diabetes, and cerebral hypoperfusion resulting from coronary heart disease can collectively increase the burden of white matter hyperintensities, which are considered to be associated with decreased cognitive ability and increased risk of depression, and the increase in their volume may reflect cerebrovascular lesions and neurodegenerative changes [32].

#### Bidirectional Pathogenic Role of Depressive Symptoms

This study clearly identifies depressive symptoms as an independent risk factor for moderate-to severe-cognitive impairment, with a negative correlation between the two (r = –0.552). On one hand, depressive states can lead to excessive activation of the hypothalamic–pituitary–adrenal axis, resulting in sustained elevation of cortisol, which in turn impairs hippocampal neurogenesis. A study by Papakokkinou and Ragnarsson [33] showed that high cortisol levels can reduce hippocampal volume and shrink the amygdala and prefrontal cortex, thereby affecting spatial memory ability. On the other hand, the decline in abilities of daily living caused by cognitive impairment can exacerbate psychological stress. As Teixeira *et al*. [34] found, executive dysfunction can lead to poorer treatment outcomes in patients with late-onset depression.

#### Regulatory Role of Social Support and BMI

This study found that an MSPSS score ≥52.5 points was a risk factor for cognitive impairment, suggesting that low social support exacerbates cognitive decline. This is similar to the conclusion of Ma *et al*. [35], whose study shows that high-level social support can reduce the risk of cognitive impairment in the elderly. In elderly patients with multiple comorbidities, reduced social interaction and insufficient emotional support may induce neuroinflammation, thereby accelerating cognitive decline. Possible mechanisms include insufficient cognitive stimulation due to reduced social interaction, and chronic stress caused by lack of emotional support—both of which jointly promote neuroinflammatory responses. The finding that BMI ≥21.42 kg/m^2^ becomes a risk factor contradicts the traditional view that obesity increases cognitive risk. This may be due to the coexistence of pathological obesity and sarcopenia caused by chronic diseases. The damaging effect of insulin resistance and elevated inflammatory factors (such as interleukin-6 and tumor necrosis factor–α) induced by this coexistence on cognition outweighs the risk of nutritional deficiency associated with low BMI [36, 37]. Our review of the original NRS-2002 data revealed that 37.86% of patients with a BMI ≥21.42 kg/m^2^ exhibited nutritional risk (compared with 22.13% in the BMI <21.42 kg/m^2^ group, *p* < 0.05), suggesting that underlying malnutrition may be the cause of this abnormal association.

### Study Limitations

This study has the following limitations. First, the single-centre cross-sectional design cannot clarify the causal relationship. For example, the sequential order of the bidirectional effect between depression and cognitive impairment still needs to be verified by cohort studies. Second, the sample size is relatively limited—especially the severe cognitive impairment group, which only includes 7 cases—this may affect the stability of stratified analysis. In the future, it is necessary to expand the sample size and refine the classification of cognitive impairment. Third, neuroimaging and blood biomarker data (such as white matter hyperintensity and Aβ protein) were not included, so the mechanism cannot be further explained from the pathophysiological level. Fourth, the impact of drug factors was not considered; some antihypertensive drugs and hypoglycaemic drugs may have potential effects on cognition or mood, which need to be controlled in subsequent studies. In addition, this study did not distinguish between subtypes of depressive symptoms. Different depressive dimensions, such as anxious depression and somatic depression, may have different impacts on cognition, which is also a research direction for the future.

Furthermore, we did not conduct subgroup analyses based on comorbidity patterns of chronic diseases. Given that this study included four chronic diseases (hypertension, diabetes, coronary heart disease, and COPD), stratification according to specific comorbidity patterns would generate multiple subgroups, resulting in insufficient sample sizes for each subgroup, which would compromise the statistical power and reliability of the analysis. Consequently, we were unable to further investigate the effects of different comorbidity patterns on cognitive dysfunction. Future studies are recommended to expand the sample size and conduct in-depth subgroup analyses based on comorbidity patterns of chronic diseases to clarify the relevant associations.

## Conclusions

This study confirms that in elderly patients with chronic underlying disease, the more severe the cognitive impairment, the more obvious the patient’s depressive symptoms. The number of chronic diseases, GDS-15 score, low social support, and high BMI are independent risk factors for moderate-to-severe cognitive impairment. Key links connecting these factors to cognitive impairment may include chronic disease burden, neuroinflammation, hypothalamic–pituitary–adrenal axis overactivation, and nutritional disorders. These findings provide a clinical basis for the mechanism research of cognitive–emotional comorbidity and also lay a foundation for formulating targeted intervention strategies. Future studies should adopt a multi-centre cohort design, combine neuroimaging and molecular biology technologies to explore the pathological pathway of “chronic comorbidity → neural network abnormalities → cognitive-emotional comorbidity”. At the same time, intervention studies should be carried out to verify the effect of emotional management combined with cognitive training on delaying cognitive decline and ultimately improve the quality of life of elderly patients with chronic diseases.

## Availability of Data and Materials

All experimental data included in this study can be obtained by contacting the corresponding author if needed.
